# Post-Traumatic Stress Disorder (PTSD) and Cardiovascular Diseases: A Systematic Review and Meta-Analysis

**DOI:** 10.3390/jcm14227979

**Published:** 2025-11-11

**Authors:** Omar Anwar Saleh Al Nakhebi, Raluka Albu-Kalinovic, Oana Neda-Stepan, Catalina Giurgi-Oncu, Cătălina-Angela Crișan, Virgil-Radu Enatescu, Ileana Marinescu

**Affiliations:** 1 Doctoral School, “Victor Babes” University of Medicine and Pharmacy, 300041 Timisoara, Romania; omar.alnakhebi@umft.ro (O.A.S.A.N.); raluka.kalinovic@umft.ro (R.A.-K.); oana.neda-stepan@umft.ro (O.N.-S.); 2 Department of Neurosciences, “Victor Babes” University of Medicine and Pharmacy, 300041 Timisoara, Romania; catalina.giurgi@umft.ro; 3 Department of Neurosciences, Iuliu Hatieganu University of Medicine and Pharmacy, 400347 Cluj-Napoca, Romania; ccrisan@umfcluj.ro; 4 Faculty of Medicine, University of Medicine and Pharmacy of Craiova, 200349 Craiova, Romania

**Keywords:** Post-Traumatic Stress Disorder (PTSD), cardiovascular diseases

## Abstract

**Objective:** This meta-analysis aimed to examine the bidirectional association between PTSD and cardiovascular disease (CVD) by evaluating the following: (1) the risk of increased CVD incidence in individuals with PTSD; and (2) the prevalence of PTSD in patients with cardiovascular disease. **Methods:** Using the Preferred Reporting Items for Systematic Reviews and Meta-Analyses (PRISMA) guidelines, a literature search was conducted of the PubMed/Medline, Scopus, and Web of Science databases without using a temporal publication range. For the PTSD-to-CVD direction, 18 studies were combined. For the CVD-to-PTSD direction, 11 studies that ascertained the incidence or prevalence of PTSD following a CVD event were combined. **Results:** The findings confirm the bidirectional and clinically significant relationship between CVD and PTSD. **Conclusions**: These data underscore the need to integrate trauma-informed approaches into cardiovascular care and stress management into psychiatric treatment to stop this pathological cycle.

## 1. Introduction

Post-Traumatic Stress Disorder (PTSD) is a complex psychiatric disorder consequent from extremely traumatic experiences or threatening events, ranging from warfare and natural disasters to scenarios involving death or risk of death, serious injury, or sexual violence of the patient or loved ones. This mental health condition, according to the Diagnostic and Statistical Manual of Mental Disorders, Fifth Edition (DSM-IV; American Psychiatric Association, 2013), includes in its symptomatic frame intrusive re-experiencing of the traumatic event (e.g., flashbacks, nightmares), persistent avoidance of trauma-related thoughts, feelings, or external reminders, negative alterations in cognition and mood, and marked alterations in arousal and reactivity, including hypervigilance and irritability [1]. While worldwide data report that over 70% of the population reported a traumatic event, the prevalence rate of PTSD was reported around 3% in the general population, with cross-national variation according to exposure to traumatic experiences (e.g., war, natural disasters), vulnerability factors (e.g., psychiatric condition), and sociodemographic correlates (e.g., age, gender, socioeconomical status) [2,3,4].

Given its notable prevalence in the general population and the substantial burden it imposes on mental health, PTSD has increasingly been recognized not only as a psychiatric disorder but also as a condition with wide-ranging implications for physical health, defining it as a multisystemic condition. Accordingly, in recent years, growing evidence has emphasized that the impact of PTSD extends beyond psychological suffering, involving significant alterations to biological systems. Increasing attention has been directed to the somatic consequences of chronic post-traumatic stress responses, including their impact on cardiovascular functioning [5,6].

Among these, cardiovascular disease (CVD) has emerged as one of the most consistently associated medical outcomes in PTSD, providing the rationale for investigating PTSD not only as a consequence of trauma but also as a relevant clinical factor in the development and progression of chronic physical diseases [7].

In this frame, a large amount of the literature focused on exploring the association of PTSD with cardiovascular conditions, which suggests a bidirectional relationship between PTSD and cardiovascular disease (CVD). On one hand, PTSD has been consistently linked with incident CVD, Coronary Artery Disease (CAD), myocardial infarction (MI), and stroke. In their comprehensive meta-analysis, Padhi et al. demonstrated that individuals with Post-Traumatic Stress Disorder (PTSD) exhibit a significantly elevated risk for cardiovascular disease (CVD), myocardial infarction (MI), and stroke, underscoring the physiological toll of chronic psychological distress. Specifically, the authors reported that PTSD increases the risk for any CVD by 42% (HR 1.417, 95% CI 1.313–1.522), MI by 42% (HR 1.415, 95% CI 1.331–1.500), and stroke by over 100% (HR 2.074, CI 1.165–2.982) [8]. Similar data emerged by previous synthesis on coronary heart disease (HR 1.61, 95% CI 1.46–177) [9]. Most relevant to data emerging from these studies is robust association, even after the adjustment for other cardiovascular risk factors (e.g., age, hypertension, body mass index).

On one side, the higher risk for cardiac events associated with PTSD may be ascribed to the feature of this psychiatric condition that is characterized by a chronic dysregulation of the autonomic nervous system (ANS), including persistent sympathetic hyperactivity and parasympathetic withdrawal, as well as hypothalamic–pituitary–adrenal (HPA) axis dysfunction [10,11]. This autonomic imbalance predisposes patients to hypertension, tachycardia, impaired baroreflex sensitivity, endothelial dysfunction, and increased myocardial workload—all antecedents of HF pathogenesis [10,11,12,13,14]. Alterations in HPA axis, reduced basal cortisol, heightened cortisol receptor sensitivity, elevated corticotropin-releasing hormone levels, and increased catecholamine secretion, which fuel chronic allostatic load, contribute to metabolic dysregulation (e.g., insulin resistance, visceral adiposity) and related effects on vascular and cardiac health [15].

Behavioral and lifestyle pathways further interconnect PTSD and HF: individuals with PTSD exhibit disproportionately higher prevalence of smoking, sedentary behavior, poor dietary habits, and medication non-adherence, all of which accentuate risk for hypertension, dyslipidemia, obesity, and eventual HF [16]. Socioeconomic adversity commonly experienced in PTSD can further amplify these disparities in cardiovascular risk. Collectively, PTSD creates a confluence of biological, behavioral, and social determinants that may drive HF onset and progression.

This complex interplay between physiological dysregulation, maladaptive health behaviors, and contextual vulnerabilities highlights the multifactorial nature of the association between PTSD and cardiovascular outcomes. However, while much of the current literature has focused on PTSD as a precipitating risk factor for cardiovascular disease, emerging findings also suggest that the onset of major cardiac events—such as myocardial infarction or Coronary Artery Disease—can act as severe, life-threatening stressors capable of triggering post-traumatic responses and, in some cases, full-blown PTSD [7,16]. This reciprocal influence underscores the need to examine the bidirectional relationship between psychological trauma and cardiovascular disease, moving beyond a unidirectional risk framework and toward a more integrated biopsychosocial understanding.

The study proposes to assess the bidirectional nature of Post-Traumatic Stress Disorder (PTSD) and cardiovascular disorders (CVSDs).

The proposed meta-analysis will thus synthesize evidence for both directional pathways: (1) quantifying the role of PTSD in the onset and progression of CVD and CAD, in terms of risk ratio, with the hypothesis to confirm the data reported by previous systematic review, and (2) examining the incidence for PTSD among individuals with cardiovascular events, with the aim to implement evidence of the bidirectional relationship between trauma and heart health.

## 2. Materials and Methods

This systematic review was conducted according to the Preferred Reporting Items for Systematic Reviews and Meta-Analyses (PRISMA) meta-analysis guidelines [17].

The protocol for this review was prospectively registered in the PROSPERO (International Prospective Register of Systematic Reviews) database under registration number CRD420251153868. Available online https://www.crd.york.ac.uk/PROSPERO/view/CRD420251153868 (accessed on 30 September 2025).

### 2.1. Eligibility Criteria

All studies investigated the association in human populations between Post-Traumatic Stress Disorder (PTSD) and cardiovascular disease (CVD), including Coronary Artery Disease (CAD), stroke, Heart Failure (HF), Angina Pectoris (AP), myocardial infarction (MI), and Ischemic Heart Failure (IHF). All studies belonging to the categories of observational studies (cohort, case–control, cross-sectional) and interventional studies were included if they reported reliable information to explore the risk ratio and proportion of the sample with the characteristics subject to investigation in this work (participants with PTSD condition and with cardiovascular diagnosis).

No restrictions were placed on the publication timeframe, and studies involving patients of any age were considered.

Studies were excluded if they were not published in English, or if they were reviews, meta-analyses, editorials, case reports, or animal studies. Also, studies with missing data were excluded; however, when necessary and possible, study’s authors were contacted to clarify missing or ambiguous data before the exclusion of it.

### 2.2. Information Sources and Search Strategy

A comprehensive systematic search was conducted across the PubMed, Scopus, and Web of Science databases, selecting original research studies published in English without using a temporal publication range. The search combined controlled vocabulary and keywords related to PTSD (e.g., “Post-Traumatic Stress Disorder,” “PTSD,” “Trauma”) and CVD (e.g., “Cardiovascular Disease”), using Boolean operators to maximize sensitivity. The specific script is reported in Table 1. Additionally, reference lists of included studies and relevant reviews were screened to identify further eligible studies.

### 2.3. Selection Process and Data Collection Process

Two independent reviewers (O.A.S.A.N. and V.-R.E.) screened all titles and abstracts to identify eligible studies according to the eligibility criteria. Full-text articles were subsequently retrieved and assessed for inclusion independently by the same reviewers. Disagreements were resolved through discussion or consultation with a third senior reviewer to ensure impartial decision-making (I.M.). No automation tools were used during screening, and all translations (when needed) were performed by qualified personnel to avoid language bias.

Data extraction was carried out independently by two reviewers (O.A.S.A.N and V.-R.E.) using a standardized, pilot-tested form in Microsoft Excel. The following data were considered for each study: author, year, country, population demographics (sample size, age, sex), study design, PTSD diagnostic criteria, CVD outcomes, and key findings.

### 2.4. Data Items

The primary outcome of interest was the association between PTSD and the incidence or prevalence of CAD. Secondary outcomes included severity of CAD, cardiovascular events, and mortality related to CAD in PTSD populations. Where studies reported multiple time points or measures, all relevant data were extracted and considered.

### 2.5. Risk of Bias Assessment

Methodological quality and risk of bias were independently evaluated by two reviewers using the Newcastle–Ottawa Scale (NOS) for observational studies [18]. The NOS assesses studies based on Selection, Comparability, and Outcome domains, awarding up to nine stars. Scores were categorized as low risk of bias (7–9 stars), moderate risk (4–6 stars), or high risk (0–3 stars). Disagreements were resolved by discussion with a third reviewer. No automated bias assessment tools were used.

### 2.6. Meta-Analytic Approach

The meta-analysis was conducted via Jamovi (2.3.31.0 version).

For the studies exploring risk ratio for CVD in PTSD, the log risk ratio as the primary effect size was adopted. A random-effects model was applied to account for potential variability across studies. The degree of heterogeneity (tau^2^) was estimated using the DerSimonian–Laird method [19]. To further assess heterogeneity, both Cochran’s Q statistic [20] and the I^2^ index were computed. Whenever heterogeneity was detected (i.e., tau^2^ > 0), a 95% prediction interval for the distribution of true effect sizes was also calculated. To identify potentially influential or outlier studies, both studentized residuals and Cook’s distances were examined. Studies were flagged as potential outliers if their studentized residuals exceeded the Bonferroni-adjusted threshold based on the standard normal distribution (two-sided alpha = 0.05, adjusted for the number of studies). A study was considered influential if its Cook’s distance was greater than the median plus six times the interquartile range of all Cook’s distances.

The proportions analysis considering the frequency of the event (i.e., PTSD) in the clinical sample (i.e., population with CVD) was conducted using the random-effects model to account for between-study variability. Proportions from each study were transformed and pooled to estimate the overall effect size, reported with 95% confidence intervals. Heterogeneity was assessed using the Q statistic, the I^2^ index, and Tau^2^. Potential publication bias was examined through Rosenthal’s failsafe N, Kendall’s rank correlation test, and Egger’s regression test for funnel plot asymmetry.

All study-level estimates were computed and analyzed on the logarithmic (natural log) scale, as this transformation stabilizes variances and ensures approximate normality of sampling distributions, which is required for random-effects meta-analysis. The pooled log risk ratio (log RR) and its 95% confidence and prediction intervals were therefore reported in log units. For clinical interpretability, the corresponding risk ratio (RR) can be obtained by back-transformation using the natural exponential, according to the following relation: RR = exp (log RR). All statistical tests were two-tailed, and significance was set at *p* < 0.05.

## 3. Results

### 3.1. Study Selection

**Figure 1:**
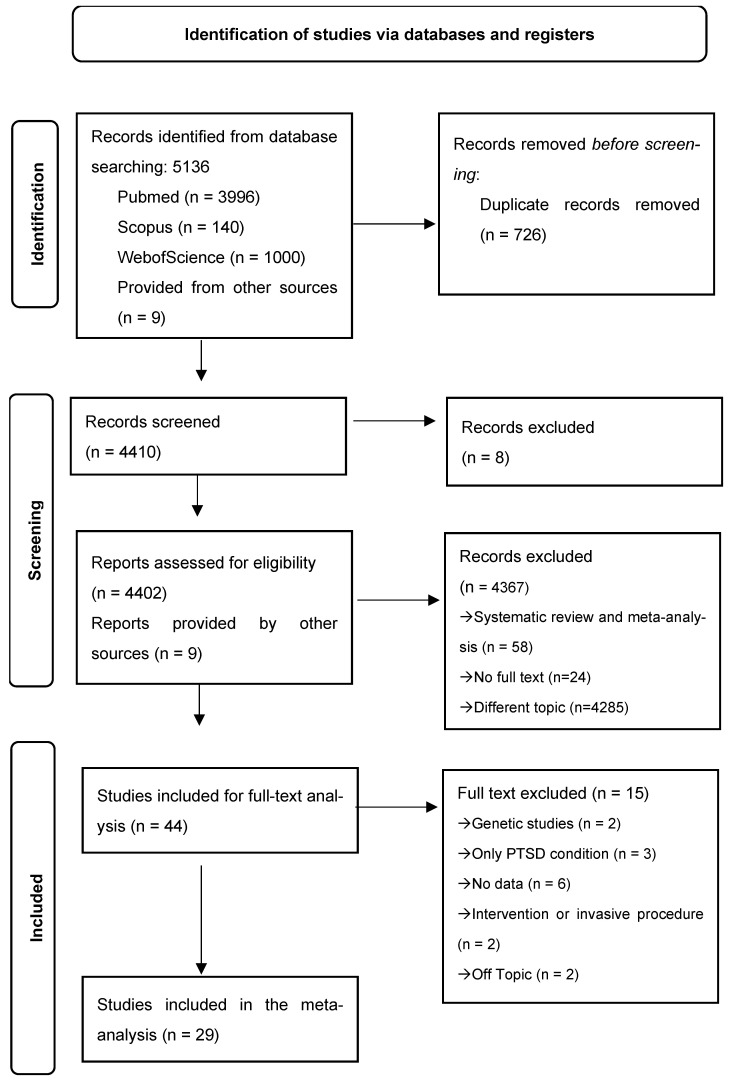
Prisma flow-chart.

The initial systematic search across PubMed, Scopus, and Web of Science databases identified a total of 5136 records. After removing duplicates (726 records), 4410 titles and abstracts were screened for relevance. Of these, 4367 records were excluded based on title and abstract screening due to not meeting the predefined inclusion criteria. Subsequently, 44 full-text articles were retrieved and assessed for eligibility. Following a detailed review (Figure 1) a final pool of 29 studies was included in this meta-analytic study. Eighteen studies included a comparison between PTSD and non-PTSD samples in terms of cardiovascular condition, while eleven studies analyzed the risk for PTSD diagnosis in samples of individuals with CVD.

### 3.2. Quality Assessment Results

Out of the 29 studies evaluated, 21 were classified as low risk of bias (L), with a score between 7 and 9 stars; 7 studies were rated as moderate risk (M), with scores ranging from 4 to 6 stars; and 1 studie were deemed high risk of bias (H), having received a score of 3 stars or lower. The distribution of quality scores suggests that the majority of included studies were of moderate to high methodological quality, thus limiting the overall risk of bias in this review (Table 2).

### 3.3. Risk Ratio for CVD in PTSD

#### 3.3.1. Characteristics of the Included Studies

The total sample of the 18 studies exploring the risk ratio included approximately 2,555,992 participants from different cohorts for retrospective or perspective studies. A total of 12 out 18 studies (67%) were carried out in the USA. The percentage of males ranged from 35 [35] to 100 [20,26,28,30,45,46]. The large amounts of males may be ascribed to the sample types. In fact, 50% (9 out 18) of the studies included cohorts of veterans (Table 2). The average age of the samples ranged from 30 [42] to 70 [47], which explored the risk ratio for CHD in a sample of former deportees. PTSD diagnosis was carried out mainly via interview for DSM-IV [9,20,22,23,24,33,38,46,47] and ICD-9 [25,28,32,42] criteria. Two studies [30,31] assessed PTSD with a self-reported questionnaire. Considering CVD condition, studies generally reported samples with multiple pathologies; however, Balint et al. (2016) [24] focused on CAD, Chen et al. (2015) [28] differentiated the risk for stroke and Ischemic Heart Disease, Rosman et al. (2019) [42] focused on transient ischemic attack, Fudim et al., 2018 [32] explored the risk for not specified heart conditions, while Mesa-Vieira et al. (2024) [39] specifically explored the MACE (e.g., Mayor Adverse Cardiovascular Events) (Table 3).

While, in general, the studies reported and discussed an increased risk in PTSD for CVD, three of the selected works did not confirm this association [24,33,38]. The three studies had small sample sizes (<100) and a cross-sectional design in common. However, while Balint and Lima’s study referred to the general population, Grenon included a sample of veterans [33,38].

#### 3.3.2. Quantitative Synthesis

**Figure 2:**
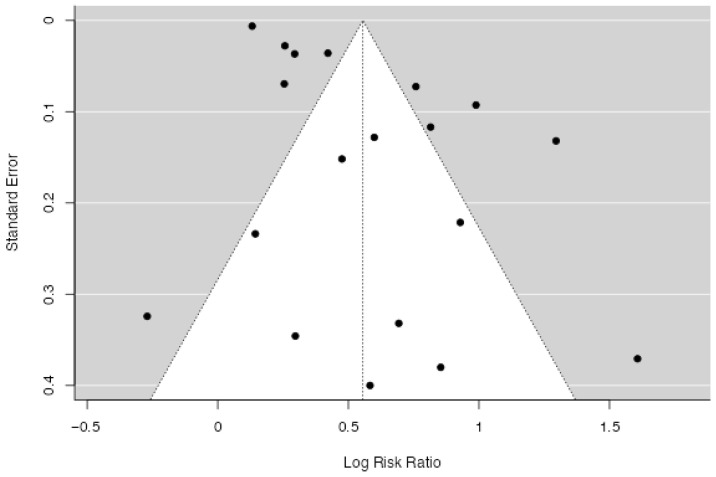
Funnel plot of the studies included for the analysis of the risk ratio for CVD in PTSD (k = 19).

**Figure 3:**
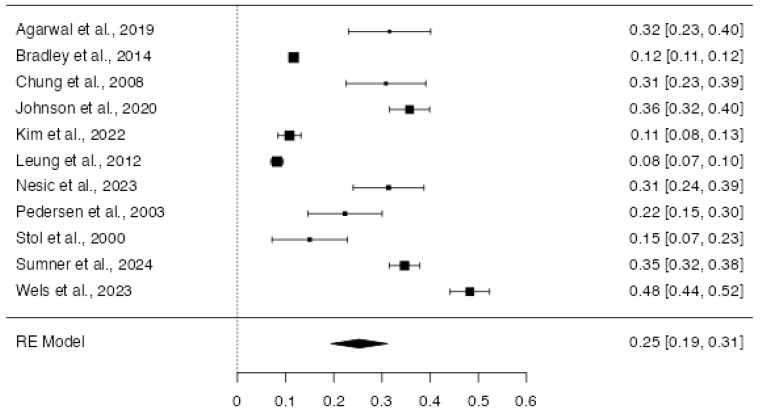
Forest plot of the studies included for the analysis of the proportion of PTSD in CVD (k = 11). This forest plot summarizes the results of multiple studies. Each line represents a single study, showing its estimated effect and 95% confidence interval. The size of each square reflects the weight of the study in the overall analysis. The diamond at the bottom represents the combined effect across all studies [21,27,29,34,36,37,40,41,43,44,48].

**Figure 4:**
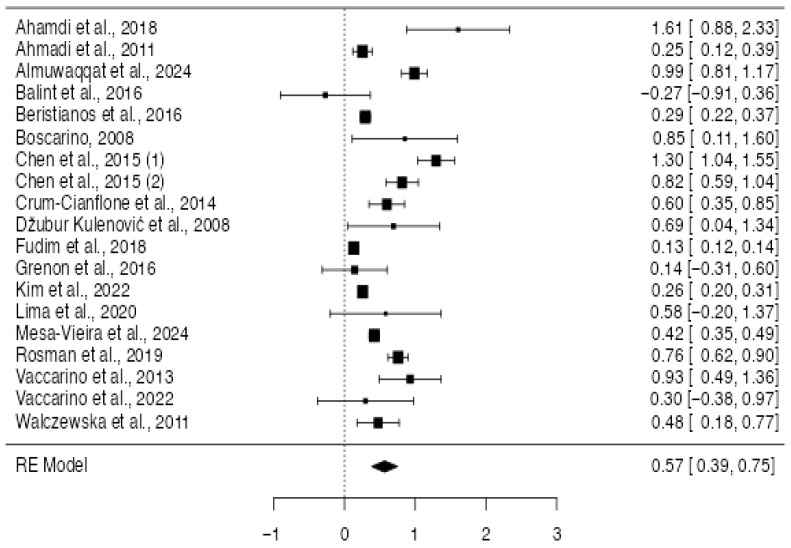
Forest plot of the studies included for the analysis of the risk ratio for CVD in PTSD (k = 19). This forest plot summarizes the results of multiple studies. Each line represents a single study, showing its estimated effect and 95% confidence interval. The size of each square reflects the weight of the study in the overall analysis. The diamond at the bottom represents the combined effect across all studies [9,22,23,24,25,26,28,30,31,32,33,35,38,39,42,45,46,47].

The analysis included 19 studies (18 primary studies and one, divided into two different outcomes). The observed log risk ratios ranged from −0.270 to 1.60. Notably, 95% of the estimates were positive (see forest plot). The overall log risk ratio was 0.56 with a 95% confidence interval ranging from 0.423 to 0.688, indicating a statistically significant effect (z = 8.22, *p* < 0.0001; Figure 2). Heterogeneity across studies was substantial, as shown by the Q statistic (Q(18) = 407.05, *p* < 0.0001), a tau^2^ value of 0.0, and an I^2^ of 95.58%. The prediction interval (0.07 to 1.05) suggests that, despite heterogeneity, most true effects are likely in the same direction as the average effect (Figure 3). The study of Chen et al. had a studentized residual above the cutoff value (±3.01), suggesting it may be an outlier [28]. However, no studies were deemed highly influential based on Cook’s distances. Finally, the regression test for funnel plot asymmetry was significant (*p* = 0.04), indicating possible publication bias, whereas the rank correlation test was not (*p* = 0.24). A trim-and-fill procedure suggested two potentially missing studies, which minimally reduced the pooled effect (log RR 0.48 [95% CI 0.27–0.68] vs. 0.56 [95% CI 0.33–0.79]). The association therefore remained robust to correction for small-study effects (Figure 4).

As a further sensitivity assessment, we repeated meta-analyses after excluding studies rated as having either high or moderate risk of bias. On the log scale, the pooled effects remained directionally consistent with the main analyses (log RR = 0.617, 95% CI 0.454–0.780, Q = 393.6, I^2^ = 95.9%). These findings confirm that the main conclusions remain stable even when excluding lower-quality studies. We also examined the PTSD ascertainment method as a potential tool. Studies using the DSM-III-R showed a larger pooled effect with minimal residual heterogeneity (log RR = 0.91, 95% CI 0.53–1.28; Q = 0.03, I^2^ = 0.0%). For DSM-IV studies, the pooled effect was log RR = 0.50 (95% CI 0.15–0.84; Q = 57.61, I^2^ = 87.85%). For ICD-9, the pooled effect was log RR = 0.64 (95% CI 0.35–0.91; Q = 201.94, I^2^ = 98.02%). For ICD-10, the pooled effect was log RR = 0.34 (95% CI 0.05–0.72; Q = 211.65, I^2^ = 92.38%). These findings suggest that part of the between-study variability reflects differences in PTSD diagnostic criteria.

### 3.4. Proportion of PTSD in CVD

#### 3.4.1. Characteristics of the Included Studies

The total sample of the 11 studies exploring the risk ratio included approximately 147,825 participants from different cohorts for retrospective or perspective studies. A total of 5 out 11 studies (45%) were caried out in the USA. The percentage of males ranged from 2.5 [29] to 78 [27]. Average age ranged from 52 [29,44]) to 67 [29]. PTSD diagnosis or risk was carried out in multiple methods and according to different diagnostics criteria (Table 4). Mainly, self-reported measures were adopted, except for 3 out 11 studies [27,29,36]. Considering CVD condition, they ranged from CHD [21,36] to coronary artery dissection [29,44] (Table 4).

#### 3.4.2. Quantitative Synthesis

**Figure 5:**
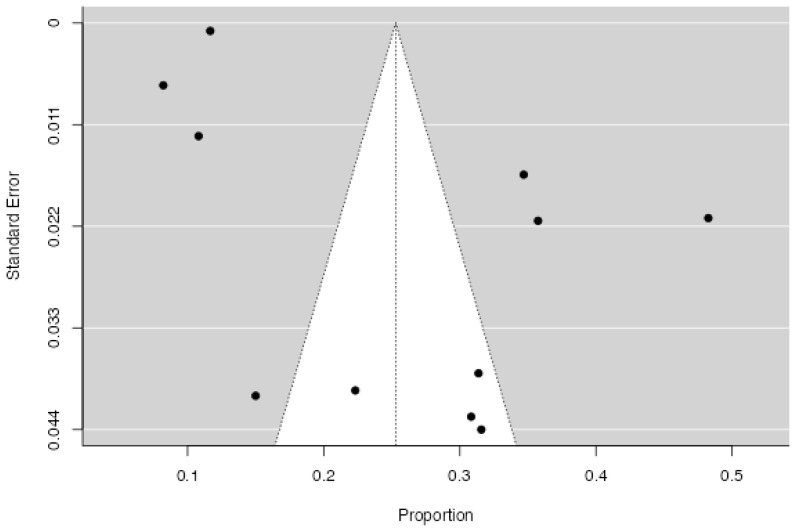
Funnel plot of the studies included for the analysis of the proportion of PTSD in CVD (k = 11).

The random-effects meta-analysis conducted to estimate the overall proportion across the 11 included studies reported a pooled proportion of 0.25 (*SE* = 0.03), indicating that approximately 25.3% of the target population met the condition of interest. This estimate was statistically significant (*Z* = 8.40, *p* < 0.001), with a 95% confidence interval ranging from 0.19 to 0.31 (Figure 3). There was substantial heterogeneity among the studies, as indicated by an *I*^2^ value of 98.65%, suggesting that nearly all of the variability in effect sizes was due to true differences across studies rather than sampling error. The between-study variance (*Tau*^2^) was estimated at 0.009 (*SE* = 0.009), and the *Q* statistic was significant (*Q*(10) = 738.92, *p* < 0.001), further confirming the presence of heterogeneity. Publication bias was assessed using Rosenthal’s failsafe *N*, which indicated that 23,521 missing null studies would be required to bring the observed effect to non-significance (*p* < 0.001), suggesting the robustness of the findings. Neither the rank correlation test (Kendall’s τ = 0.20, *p* = 0.45) nor the regression test for funnel plot asymmetry (*Z* = 1.65, *p* = 0.100) provided evidence of significant publication bias (Figure 5). As a further sensitivity assessment, we repeated meta-analyses after excluding studies rated as having either high or moderate risk of bias. The pooled effects remained directionally consistent with the main analyses (logit = −1.127, 95% CI −1.820–−0.435, Q = 1227.3, I^2^ = 99.4%; excluded studies: [27,34,36]. These findings confirm that the main conclusions remain stable even when excluding lower-quality studies. To ensure robustness, a sensitivity meta-analysis was conducted excluding studies at high or moderate risk of bias. On the logit scale, the pooled PTSD prevalence of 0.22 was noted (95% CI 0.14–0.33%). These results confirm that the exclusion of lower-quality and potentially influential studies does not materially alter the overall conclusions. Given the high variability of the data in this analysis, no subgroup analyses were conducted.

## 4. Discussion

This meta-analysis significantly enhances the understanding of the complex, bidirectional relationship between cardiovascular disease (CVD) and Post-Traumatic Stress Disorder (PTSD), helping in quantifying (i) the increased risk of incident CVD in individuals with PTSD and (ii) the occurrence of PTSD in individuals with cardiovascular conditions. These two aspects inform various, yet connected, clinical and pathophysiological processes and strengthen the case for an integrative biopsychosocial framework.

A primary result, according to the first aim of this work, suggests that PTSD increases the risk of developing CVD by 75% compared to controls who do not experience PTSD symptomatology. This result is in line with recent studies that focused on different CVD conditions and substantiated the hypothesis that chronic psychological trauma induces a cascade of biological alterations deleterious to cardiovascular well-being, with an increase in cardiac mortality, even after adjusting for numerous demographic, socioeconomic, and traditional cardiovascular risk factors [8,49]. Moreover, the inclusion of more studies in our work broadens the scope and enhances the robustness of the findings reported by previous studies.

This direction of the relationship, which predicts an increase in cardiovascular risk in psychopathological conditions, provides interesting insights into the relationship between physiological and psychological aspects. Neurobiologically, the changes characterizing PTSD symptoms and occurring in the autonomic system may affect central autonomic network functions, such as brainstem nuclei, the amygdala, insula, and the prefrontal cortex—regions that, if altered, disrupt homeostatic feedback, elevating cardiovascular stress [16,50,51]. Recent research highlights how autonomic dysfunction in PTSD, measured, for example, through reduced heart rate variability (HRV), is an independent predictor of negative cardiovascular outcomes, signaling a persistent state of sympathetic hyperarousal that overloads the cardiovascular system [16,52]. Similarly, HPA axis dysregulation typically affecting PTSD is linked with disturbances in metabolism such as insulin resistance and fat deposition in visceral areas, as a further risk factor for heart health [52,53]. The role of chronic inflammation is increasingly central; recent studies demonstrate that the persistent elevation of inflammatory biomarkers such as high-sensitivity C-reactive protein (hs-CRP) and various pro-inflammatory cytokines (e.g., IL-6, TNF-α) partially mediates the association between PTSD and CVD, reflecting a constant immune activation that damages the vascular endothelium [54]. The studies that explored this association also reported the role of modifiable risk factors such as physical inactivity, smoking and alcohol consumption, and poor diet, which have been shown to be more common in people with PTSD than in other populations [55,56]. Socioeconomic adversity, common in PTSD, also limits access to preventive care, further increasing cardiovascular risk [26]. However, the presence of these risk factors only partially explains the link between PTSD and heart disease, and further studies should explore what and how it influences the risk ratio confirmed by this meta-analysis. Of specific interest, three of our included studies were not able to detect significant risk increases; they may have been weakened by their small numbers and cross-sectional study design, emphasizing the importance of strong longitudinal data to answer questions of causal inference [24,33,38].

The second key finding of this meta-analysis, in line with the second objective of the study, demonstrates that approximately one-quarter of individuals with CVD meet the criteria for PTSD or exhibit clinically significant PTSD symptoms. This prevalence markedly exceeds general population estimates (~3–7%), emphasizing the psychological burden imposed by cardiovascular events [7,44]. The findings of the present meta-analysis indicate that approximately one in four individuals (25.3%) develop Post-Traumatic Stress Disorder (PTSD) following a cardiovascular event. This prevalence is substantially higher than that observed in the general population, where PTSD rates typically range from 3% to 5%, with some variation according to cultural and social context [3,56]. The prevalent data reported by the included studies confirmed the considerable psychological impact of acute cardiac episodes. The consistency of this estimate across studies, despite substantial heterogeneity (I^2^ = 98.65%), underscores the robustness and clinical relevance of the phenomenon, further supported by the absence of significant publication bias. The traumatic nature of cardiac events, such as myocardial infarction, stroke, or coronary artery dissection, which individuals may perceive as life-threatening, can precipitate PTSD through mechanisms analogous to other trauma exposures [57]. Neurobiologically, such acute stressors activate limbic and brainstem circuits mediating fear and stress responses, triggering maladaptive neuroplastic changes [58]. Additionally, the physical sequelae of CVD (e.g., chronic pain, functional impairment) may serve as a trauma reminder, sustaining PTSD symptomatology [7]. Importantly, such events impact cognitive representation of illness, with an impact on loss of control, and perceived helplessness that affects trauma processing (APA, 2013). This finding reinforces the need to conceptualize PTSD not only as a risk factor for cardiovascular disease (CVD) but also as a potential outcome of cardiac trauma, in line with the emerging bidirectional model of mind–heart interactions [7,8,13,14]. The cardiovascular system, under acute threat, becomes both the site of pathology and the trigger of psychological sequelae. In this light, the bidirectionality is not merely sequential but reciprocal: physiological dysregulation inherent in cardiac disease may contribute to PTSD onset via sustained autonomic arousal, inflammatory activation, and neuroendocrine imbalance, while PTSD may in turn exacerbate cardiovascular vulnerability through both behavioral (e.g., sleep disruption, poor adherence) and biological pathways (e.g., sympathetic overdrive, HPA axis dysfunction) [59]. If we consider that PTSD is associated with poorer adherence to cardiac rehabilitation, reduced physical activity, and heightened inflammatory markers, all of which predict worse cardiovascular prognosis, it appears relevant to consider this association for preventing negative outcomes for health. PTSD and CVD comorbidity complicates risk stratification and management, as PTSD symptoms may mask or exacerbate somatic complaints, requiring integrated care approaches [60,61,62]. Despite the known prevalence of psychological distress in cardiac populations, PTSD is often underdiagnosed in cardiology settings due to symptom overlap (e.g., chest tightness, tachycardia), stigma, or insufficient mental health training among medical personnel [6]. This oversight is clinically relevant, as cardiac-induced PTSD has been associated with poorer rehabilitation outcomes, increased re-hospitalization, and elevated mortality risk [60]. Considering these findings, we argue for a reconceptualization of post-cardiac care that incorporates trauma-informed approaches, routine PTSD screening (e.g., PCL-5), and early psychological intervention—especially in high-risk patients. Moreover, future studies should adopt longitudinal designs to disentangle the temporal dynamics of PTSD onset following cardiac events, explore gender-specific vulnerability (e.g., women may report higher post-MI PTSD rates), and evaluate the efficacy of integrated psychosomatic interventions.

Taken together, these findings support a dynamic, bi-directional relationship between PTSD and CVD. PTSD not only potentiates vulnerability to cardiovascular disease through neuroendocrine, autonomic, and behavioral pathways but also presents as a common sequel to cardiac events and offers a cycle of physical and psychological morbidity. This exchange points to the fact that cardiovascular disease and trauma are reciprocally reinforcing conditions that need to be jointly evaluated and treated across disciplines. Also, the extreme heterogeneity across studies, differences in PTSD diagnostic assessment, and multiple cardiovascular outcomes suggest the need for standardized approaches to enhance effect estimates and test moderating variables [63]. Future studies will require repeated longitudinal, neurobiological, and behavioral assessments to disentangle temporal relationships and identify therapeutic targets [64,65].

### Limitations and Further Direction

Despite the robust findings of this meta-analysis, several limitations must be taken into account. First, there was significant heterogeneity between studies in both the PTSD-to-CVD risk and the proportion of PTSD in CVD samples. This heterogeneity likely reflects differences in study design (prospective vs. retrospective), sample (veterans vs. general population), PTSD ascertainment methods (clinical interview vs. self-report questionnaire), and cardiovascular outcome definitions (total CVD vs. Coronary Artery Disease or stroke alone). In this study, heterogeneity was explored specifically in relation to the method of PTSD diagnosis. Our subgroup analysis showed that studies using clinical or structured diagnostic approaches (e.g., DSM-III-R) highlighted larger and more consistent associations with cardiovascular outcomes, whereas those relying on alternative diagnostic criteria exhibited greater variability. These findings highlight the impact of ascertainment methods on pooled estimates and emphasize the need for standardized diagnostic criteria for PTSD to enable more reliable comparisons and synthesis of evidence across studies. Although this represents a clinically relevant source of variability, additional moderators need systematic evaluation in future meta-analytic research. In addition, although minor asymmetry was observed in the publication bias tests, sensitivity analyses using the trim-and-fill method indicated that the potential influence of missing studies was minimal, confirming the robustness and stability of the pooled estimates. Second, the fact that most of the research is being conducted in the United States, and more particularly in veteran cohorts, may limit the external validity of research findings to other groups and health systems. Veterans may have unique trauma exposures, comorbidities, and healthcare use patterns that may not be representative of the general population. In addition, gender distinctions were evident, with most studies consisting of predominantly male samples, which restricts the understanding of PTSD-CVD associations in women, despite evidence of sex differences in PTSD expression and cardiovascular risk [13,14]. Third, although the meta-analysis was conducted on large sample sizes, the observational nature of the included studies precludes definitive causal inference. Finally, the reliance on self-reported PTSD symptoms in most studies is a measurement validity, recall bias, and misclassification concern that may overestimate or underestimate effect estimates. Future research with objective biomarkers and longitudinal measures will be necessary to disentangle mechanistic pathways and temporal relationships. Future research should (i) delineate causal mechanisms with prospective longitudinal designs and biomarker assessments; (ii) examine sex and gender differences in both directions of the PTSD–CVD association; and (iii) evaluate the effectiveness of integrated care models in mitigating the dual burden of psychological and cardiovascular illness.

## 5. Conclusions

This meta-analysis establishes an inverse, significant, and clinically significant association between PTSD and cardiovascular disease, in favor of the belief that cardiovascular events are more than medical emergencies but also hidden traumatic stressors with lasting psychological impact. Based on the results of this study and future directions, the following can be concluded: despite the longitudinal design of some included studies, the observational nature precludes causal inference, which should be explicitly recognized. PTSD patients carry a significantly higher risk of developing CVD, presumably through interactive neurobiological, behavioral, and psychosocial mechanisms. Conversely, a large percentage of patients with cardiovascular disease also share symptoms with PTSD, indicating the psychological impact of cardiac events and the necessity of comprehensive healthcare. Screening for PTSD symptoms among cardiac patients should be a priority for practitioners, and cardiovascular risk factors should be assessed in patients with PTSD. Multidisciplinary treatments for autonomic dysfunction, stress regulation, and behavioral health are promising to decrease the intertwined burden of these diseases. Future research efforts ought to enhance diagnostic approaches, elucidate sex specificity, and yield specific therapies that disrupt this pathogenic bidirectional cycle. Finally, identification and prevention of the bidirectional influence of PTSD and CVD represent a vital step toward coordinated patient care and improved long-term prognosis in both cardiovascular and mental health. From a clinical perspective, these results are of critical significance. Treatments for autonomic regulation (e.g., biofeedback, vagal nerve stimulation), HPA axis modulation, and behavioral health (e.g., trauma-focused cognitive behavior therapy, lifestyle counseling) may hold promise to interrupt this bidirectional cycle [66,67]. Incorporating mental health professional knowledge within cardiology teams may improve prognosis and quality of life among impacted patients.

## Figures and Tables

**Table 1:** Search strategy summary.

Database	Search Terms	Limits/Filters	Results Retrieved	Date of Search
PubMed	(“Post-Traumatic Stress Disorder” OR “PTSD” OR “trauma”) AND “Coronary Artery Disease”	English language only	3996	May 2025
Scopus	TITLE-ABS-KEY (“PTSD” OR “Post-Traumatic Stress Disorder” OR “trauma”) AND “Coronary Artery Disease”	English language only	140	May 2025
Web of Science	(“PTSD” OR “Post-Traumatic Stress Disorder” OR “trauma”) AND “Coronary Artery Disease”	English; article/review types	1000	May 2025

**Table 2:** Risk of bias evaluation.

Study	Selection (Max 4 Stars)	Comparability (Max 2 Stars)	Outcome (Max 3 Stars)	Risk of Bias Assessment	Risk of Bias
Agarwal et al./2019 [21]	★,★,★, N/A	★★	★,★,★	High Quality	**L**
Ahmadi et al./2011 [9]	★,★,★,★	★★	★,★,★	High Quality	**L**
Ahmadi et al./2018 [22]	★,★,★,★	★★	★,★,★	High Quality	**L**
Almuwaqqat et al./2024 [23]	★,★,★,★	★★	★,★,★	High Quality	**L**
Balint et al./2016 [24]	★, N/A, ★, N/A	★	★, N/A, N/A	Moderate Quality	**M**
Beristianos et al., 2016 [25]	★,★,★,★	★★	★,★,★	High Quality	**L**
Boscarino/2008 [26]	★,★,★,★	★★	★,★,★	High Quality	**L**
Bradley et al./2014 [27]	★,★,★, N/A	★★	★, ★, ★	High Quality	**L**
Chen et al., 2015 [28]	★,★,★,★	★★	★,★,★	High Quality	**L**
Chung et al./2008 [29]	★,★,★, N/A	★	★,★,★	High Quality	**L**
Crum-Cianflone and Jacobson, 2014 [30]	★,★,★,★	★★	★,★,★	High Quality	**L**
Dzubur Kulenović et al./2008 [31]	★,★,★, N/A	★	★, N/A, N/A	Moderate Quality	**M**
Fudim et al./2018 [32]	★,★,★,★	★★	★,★,★	High Quality	**L**
Grenon et al./2016 [33]	★,★,★, N/A	★★	★,★,★	High Quality	**L**
Johnson et al./2020 [34]	★, N/A, ★, N/A	★	★, N/A, N/A	Moderate Quality	**M**
Kim et al./2022 [35]	★, N/A, ★, N/A	★★	★, N/A, N/A	Moderate Quality	**M**
Kim et al./2022 [36]	★,★,★,★	★★	★,★,★	High Quality	**L**
Leung et al./2012 [37]	★, N/A, ★, ★	★	★,★,★	High Quality	**L**
Lima et al./2020 [38]	★,★,★, N/A	★★	★,★,★	High Quality	**L**
Mesa-Vieira et al., 2024 [39]	★,★,★,★	★★	★,★,★	High Quality	**L**
Nesic et al./2023 [40]	★, N/A, ★, N/A	★	★, N/A, N/A	Moderate Quality	**M**
Pedersen et al./2003 [41]	★,★, N/A, N/A	★	★,★,★	Moderate Quality	**M**
Rosman et al., 2019 [42]	★,★,★,★	★★	★,★,★	High Quality	**L**
Stoll et al./2000 [43]	★, ★, ★, N/A	★	★, ★, ★	Moderate Quality	**L**
Sumner et al./2024 [44]	★, N/A, ★, N/A	N/A	★, N/A	Low Quality	**H**
Vaccarino et al./2013 [45]	★,★,★,★	★★	★,★,★	High Quality	**L**
Vaccarino et al./2022 [46]	★,★,★,★	★★	★,★,★	High Quality	**L**
Walczewska et al./2011 [47]	★,★,★,★	★★	★, N/A, N/A	High Quality	**L**
Wells et al./2023 [48]	★, N/A, ★, N/A	★	★, N/A, N/A	Moderate Quality	**M**

**Table 3:** Main characteristics of the studies included in the quantitative synthesis of risk ratio of CAD in PTSD.

Study	Country	Sample Size	Type of Sample	Male Percentage	Mean Age of the Sample	PTSD-Diagnosis	Cardiovascular Condition	Main Results
Ahmadi et al., 2011 [9]	USA	637	Veterans	100	58.5	DSM-IV	CAC; Framingam Risk Score	PTSD associated with presence and severity of CAC PTSD independent predictor of mortality
Ahmadi et al., 2018 [22]	USA	246	General Population	72	61.5	DSM-IV	MACE	PTSD independent predictors of MACE
Almuwaqqat et al., 2024 [23]	USA	736	General Population	65	59	DSM-IV	Incidence of CVD	Higher level of current PTSD symptoms independently associated with increased risk of first and recurrent Heart Failure hospitalizations.
Balint et al., 2016 [24]	Germany	56	General Population	65	62.5	DSM-IV	CAD	No differences were reported in prevalence
Beristianos et al., 2016 [25]	USA	138,341	Veterans	95.9	65.9	ICD-9	CVD	PTSD higher rate of CVD
Boscarino, 2008 [26]	USA	4328	Veterans	100	-	DSM-III-R	CVD	PTSD prospectively associated with HD mortality
Chen et al., 2015 (1) [28]	Taiwan	26,085	General Population	20.9	36.6	ICD-9	Stroke	Higher incidence of stroke in PTSD condition
Chen et al., 2015 (2) [28]	Taiwan	26,085	General Population	20.9	36.6	ICD-9	IHD	Higher incidence of IHD in PTSD condition
Crum-Cianflone et al., 2014 [30]	USA	60,025	Veterans	60	34.4	Self-report	CHD	Higher incidence of CHD in PTSD condition
Dzubur Kulenović et al., 2008 [31]	Bosnia-Erzegovina	100	Veterans	100	40–50	Self-report	CAD	Chronic PTSD associated with dyslipidemia and increased risk of CAD
Fudim et al., 2018 [32]	USA	111,970	Veterans	100	66.5	ICD-9	Not Specified HC	Higher rate of CAD in PTDS condition
Grenon et al., 2016 [33]	USA	214	Veterans	99	69	DSM-IV	CAD	No differences were reported in prevalence
Kim et al., 2022 [35]	Korea	214,996	General Population	35	50	ICDI-10	CAD; hemorrhagic stroke, and cardiovascular mortality	Associations between PTSD and CVD outcomes
Lima et al., 2020 [38]	USA	303	General Population	52	50	DSM-IV	HF	No differences were reported in prevalence
Mesa-Vieira et al., 2024 [39]	USA	1,009,113	General Population	40	38.6	ICD-10	MACE	Higher incidence of MACE in PTSD condition
Rosman et al., 2019 [42]	IRAQ	987,855	General Population	-	30	ICD-9	TIA	Higher incidence of TIA in PTSD condition
Vaccarino et al., 2013 [45]	USA	562	Veteran Twins	100	42.6	DSM-III-R	AMI; CHD; ACS	PTSD was associated with greater than twice the risk of CHD over a median follow-up of 13 years
Vaccarino et al., 2022 [46]	USA	275	Veteran Twins	100	68	DSM-IV	CHD	PTSD higher rate of CHD
Walczewska et al., 2011 [47]	Polonia	150	Former Deportees (Siberia) vs. Control with no PTSD	50	70	DSM-IV	CHD	Former deportees with PTSD had higher prevalence of CHD

CAC = Coronary Artery Calcium; MACE = Mayor Adverse Cardiovascular Events; CVD = cardiovascular disease; CHD = Coronary Heart Disease; IHD = Ischemic Heart Disease; TIA = transient ischemic attack; HF = Heart Failure; AMI = acute myocardial infarction.

**Table 4:** Main characteristics of the studies included in proportion analysis of PTSD in CVD.

Study	Country	Sample Size	Male Percentage	Mean Age of the Sample	PTSD-Diagnosis	Cardiovascular Condition	Percentage of PTSD in Clinical Sample
Agarwal et al., 2019 [21]	USA	114	-	-	PCL-5	Cardiac Arrest	32
Bradley et al., 2014 [27]	USA	142,954	-	-	ICD-9 (Interview)	CAD	12
Chung et al., 2008 [29]	UK	120	78	67	DSM-IV (Interview)	AMI	31
Johnson et al., 2020 [34]	USA	512	2.5	52	PDS	Coronary Artery Dissection	36
Kim et al., 2022 [36]	USA	657	65	58.4	DSM-IV (Interview)	CHD	11
Leung et al., 2012 [37]	Toronto	1692	75	65.5	PTGI	MACE (HF; stroke; AMI)	8
Nesic et al., 2023 [40]	Germany	153	50	61	PCL-5	CAD	31
Pedersen et al., 2003 [41]	Denmark	112	70	60	PDS	AMI	22
Stoll et al., 2000 [43]	Germany	80	51	63.5	PTSS-10	Cardiac Surgery	15
Sumner et al., 2024 [44]	USA	859	6	52.3	DSM-IV Checklist	Coronary Artery Dissection	35
Wells et al., 2023 [48]	UK	572	63	60.5	IES-R	CHD	48

## Data Availability

All data supporting the findings of this study are included within the article. Additional information is available upon reasonable request from the corresponding author.
